# Icariin Attenuates Renal Injury in Streptozotocin-Induced Diabetic Rats with and Without Adenine-Induced Chronic Kidney Disease

**DOI:** 10.3390/ph19060971

**Published:** 2026-06-22

**Authors:** Raya Al Maskari, Haytham Ali, Priyadarsini Manoj, Mohammed Al Za’abi

**Affiliations:** 1 Department of Pharmacology and Clinical Pharmacy, College of Medicine and Health Sciences, Sultan Qaboos University, Muscat 123, Oman; priyadarsinimanoj@gmail.com (P.M.); zaabi@squ.edu.om (M.A.Z.); 2 Department of Animal and Veterinary Sciences, College of Agricultural and Marine Sciences, Sultan Qaboos University, Muscat 123, Oman; h.ali@squ.edu.om

**Keywords:** diabetes, chronic kidney disease, icariin, inflammation, oxidative stress

## Abstract

**Background**: Diabetes mellitus (DM) and chronic kidney disease (CKD) are major contributors to global morbidity and mortality, with disease progression being closely linked to persistent inflammation, oxidative damage, and apoptotic pathways. Icariin (ICA), a bioactive flavonoid compound isolated from *Epimedium brevicornum Maxim*, has attracted considerable interest because of its diverse pharmacological properties. We evaluated the effect of ICA on streptozotocin (STZ)-induced diabetic rats with or without adenine-induced CKD. This combined model reproduces several key structural and functional characteristics observed in human diabetic kidney disease and advanced CKD. **Methods**: Male Wistar rats were allocated to five treatment groups and followed for 35 days. Group 1 served as the untreated control and received standard chow; Group 2 was administered streptozotocin (STZ); Group 3 received STZ together with icariin (ICA); Group 4 received a combination of adenine and STZ; and Group 5 was treated with adenine, STZ, and ICA. ICA was administered at a dose of 200 mg/kg by oral gavage. Biochemical, oxidative stress and inflammatory markers were assessed. **Results**: Rats treated with STZ, with or without adenine, exhibited significant hyperglycemia, elevated plasma levels of cystatin C and indoxyl sulphate, increased urinary levels of N-acetyl-β-D-glucosaminidase (NAG) and NAG/creatinine ratio, and reduced creatinine clearance. Additionally, there were significant decreases in renalase activity and urine osmolality, significant increases in interleukins IL-1β and IL-6 and TNF-alpha levels, and a decrease in IL-10 level. Oxidative stress biomarkers were also significantly impaired in both groups, along with significant renal histopathological changes. ICA significantly ameliorated these alterations in both experimental groups. **Conclusions**: These findings demonstrate that ICA exerts renoprotective and anti-inflammatory effects in a clinically relevant model of advanced diabetic CKD. Further studies are warranted to elucidate the underlying mechanisms and determine the translational relevance of these findings.

## 1. Introduction

Diabetes mellitus (DM) is a chronic non-communicable disease that is rapidly becoming one of the most prevalent illnesses globally, with estimates suggesting that it will impact approximately 693 million adults by 2045 [1]. Global epidemiological studies estimate that chronic kidney disease (CKD) is present in at least one third of patients with diabetes, reaching up to 83.6% in some populations [2].

CKD is generally regarded as an incurable condition, and the current treatment strategies primarily aim to slow its progression and manage its complications [3]. This is achieved by non-pharmacological approaches involving physical activity, sodium restriction and reduced protein intake, combined with a treatment plan using pharmacological agents [3]. Despite advances in treatment, progression to end-stage renal disease remains a major challenge, with an estimated 2.6 million people worldwide currently relying on some form of renal replacement therapy, including dialysis or kidney transplantation [4]. Consequently, there is an increasing demand to develop novel agents that can improve the current management options of CKD and alleviate its associated complications. This is particularly important in the context of diabetes, where advanced stage CKD complicates the attainment of optimal glucose control as a result of impaired renal gluconeogenesis, and also because many of the available antidiabetic drugs are therapeutically contraindicated at this stage [5].

Herbal remedies have been used throughout human history and remain significant in modern medicine. Icariin (ICA), a flavonoid compound derived from the herb *Epimedium brevicornum Maxim*, has demonstrated a wide range of pharmacological effects in many in vivo and in vitro studies, including effects on osteoporosis, cancer, inflammation, neurodegenerative diseases, cardiovascular diseases and diabetes [6]. The anti-inflammatory action of ICA has been attributed to the regulation of various inflammatory cytokines, such as IL-1β, IL-6 and TNF-α [7]. The compound has also been shown to have antioxidant properties, protecting against hydrogen peroxide-induced oxidative injury, reducing ferric ions, and inhibiting lipid peroxidation [8,9]. Furthermore, through the activation of the endothelial nitric oxide synthase/nitric oxide (eNOS/NO) pathway, ICA has been shown to delay endothelial cell aging induced by homocysteine by reducing reactive oxygen species (ROS) levels and stimulating the phosphoinositide 3-kinase/protein kinase B/endothelial nitric oxide synthase (PI3K/Akt/eNOS) signaling pathway [9].

In diabetic rat models, ICA has been shown to improve diabetic retinopathy [10] and early diabetic nephropathy [11]. Furthermore, ICA has demonstrated renoprotective effects across multiple preclinical models. ICA reduced renal fibrosis in a unilateral ureteral obstruction mouse model [12] and in CKD rats subjected to 5/6 ablation and infarction (A/I) operation [13]. ICA also attenuated proteinuria and renal damage in rat models of glomerulonephritis [14], preserved renal function in adenine-induced CKD rats [15], and improved multiple parameters of acute kidney injury [16,17].

However, the effects of ICA in a combined model of diabetes-associated CKD have not been studied to date. The adenine-induced CKD model (typically 0.2–0.3% adenine in chow for ~4 weeks) is a widely used CKD model because it reproduces hallmark features of human CKD that include progressive decline in renal function, tubulointerstitial fibrosis, tubular atrophy, interstitial inflammation, anemia, and metabolic disturbances [18]. The combination of STZ-induced diabetes and adenine-induced CKD incorporates metabolic dysfunction and progressive renal injury, thereby providing a more clinically relevant model of advanced diabetic CKD. Therefore, this study aimed to investigate the impacts of ICA on adenine-induced CKD in streptozotocin (STZ)-induced diabetes in rats.

## 2. Results

### 2.1. Physiological Parameters

The effects of ICA on various physiological parameters in diabetic rats with or without CKD is presented in Table 1. Diabetes induction with STZ caused a significant decrease in body weight and relative kidney weight and a significant increase in water intake and urine flow. Similar changes were observed in diabetic rats with adenine-induced CKD, along with a significant increase in food intake and fecal output. Liver weight was higher in both experimental groups, but the difference was not statistically significant. Treatment with ICA significantly improved water intake and urine flow in both the diabetic group and diabetic CKD group, but had no significant impacts on the remaining parameters.

### 2.2. Blood Glucose and Renal Function Markers

Both the diabetic group and diabetic CKD group had significantly higher blood glucose levels, urea, creatinine, and phosphorus, along with significantly lower calcium levels (Table 2). Uric acid was higher in both groups compared to the control, but the difference was only significant in the diabetic group with CKD. Treatment with ICA significantly ameliorated all of these changes in both groups.

Both experimental groups also had significantly lower urine osmolality, creatinine and creatinine clearance, and significantly higher urine NAG levels and albumin/creatinine ratios (Table 3). Urine NAG/creatinine ratios were higher in both groups, but the difference was statistically significant only in the diabetic CKD group. ICA administration significantly reversed the changes in osmolality, creatinine, NAG levels, albumin/creatinine ratios, and creatinine clearance in the diabetic group, and significantly reversed the changes in osmolality, creatinine, NAG levels, NAG creatinine ratios, and creatinine clearance.

**Figure 1:**
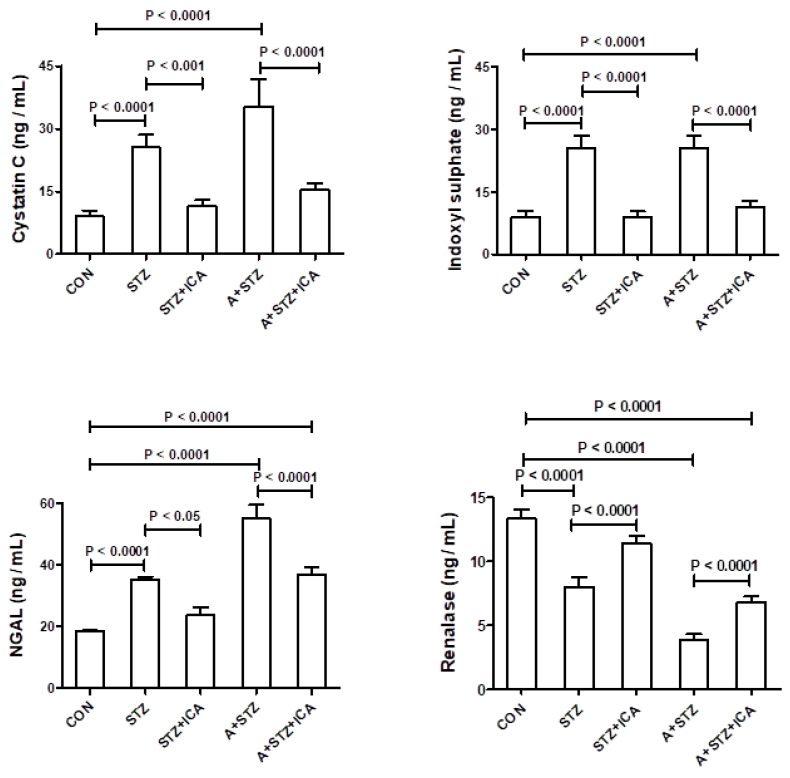
Effect of icariin (ICA) on plasma cystatin C activity, neutrophil gelatinase-associated lipocalin (NGAL) concentration, indoxyl sulphate levels, and renalase activity in control (CON) rats and rats treated with streptozotocin (STZ), with or without adenine (A). Data are presented as mean ± SEM for six animals per group (n = 6). Biomarker concentrations are expressed in ng/mL (nanograms per milliliter), with the corresponding units indicated on the respective *y*-axes. Statistical analyses were performed using one-way analysis of variance (ANOVA), followed by Bonferroni’s post hoc test for multiple comparisons. A value of *p* < 0.05 was considered statistically significant.

Furthermore, both experimental groups displayed significantly elevated plasma cystatin C, IS and NGAL levels, and significantly lower plasma renalase levels compared to the control group (Figure 1). All of these changes were significantly mitigated in the ICA-treated groups.

### 2.3. Inflammation and Oxidative Stress

**Figure 2:**
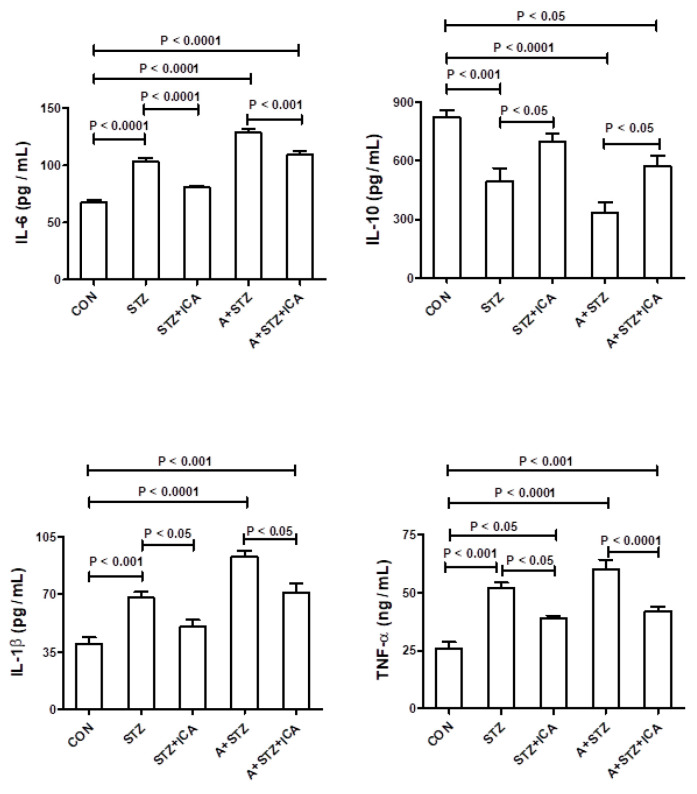
Effects of icariin (ICA) treatment on circulating levels of tumor necrosis factor-α (TNF-α), interleukin-1β (IL-1β), interleukin-6 (IL-6), and interleukin-10 (IL-10) in control (CON) rats and rats administered streptozotocin (STZ), either alone or in combination with adenine (A). Values are presented as mean ± SEM for six animals per group (n = 6). The units of measurement for each biomarker are shown on the respective *y*-axes and are reported in either ng/mL (nanograms per milliliter) or pg/mL (picograms per milliliter), as applicable. Group comparisons were performed using one-way analysis of variance (ANOVA), followed by Bonferroni’s post hoc test. Statistical significance was defined as *p* < 0.05.

**Figure 3:**
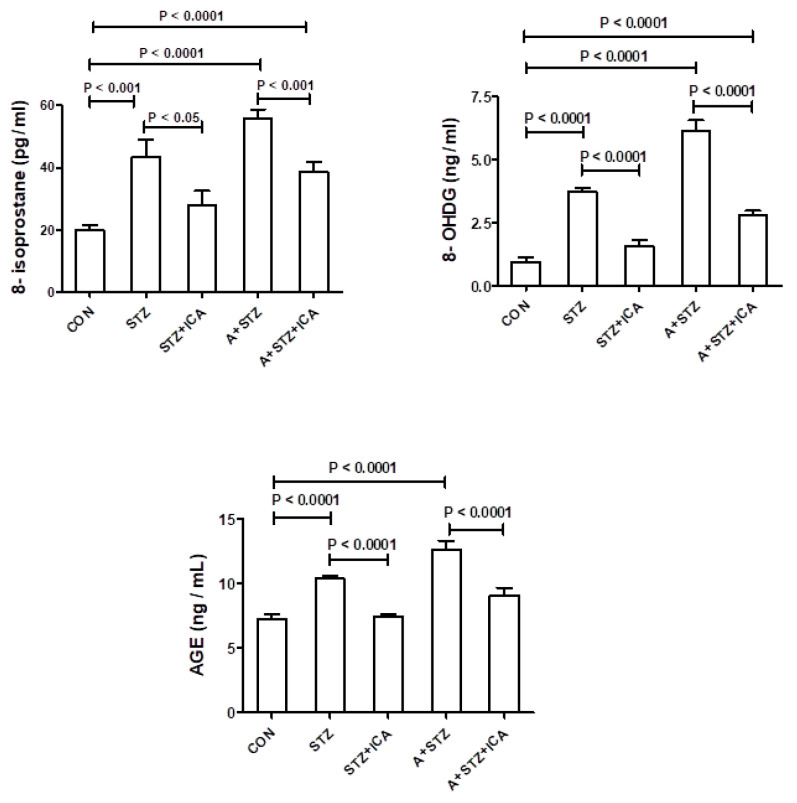
Effects of icariin (ICA) on plasma levels of 8-isoprostane, advanced glycation end-products (AGEs), and 8-hydroxy-2′-deoxyguanosine (8-OHdG) in control (CON) rats and in rats receiving streptozotocin (STZ), with or without adenine (A). Values are presented as mean ± SEM for six animals in each group (n = 6). The units used for each biomarker are displayed on the corresponding *y*-axes and reported as either pg/mL (picograms per milliliter) or ng/mL (nanograms per milliliter), depending on the analyte measured. Statistical differences among groups were evaluated using one-way analysis of variance (ANOVA), followed by Bonferroni-adjusted multiple comparisons. Statistical significance was defined as *p* < 0.05.

**Figure 4:**
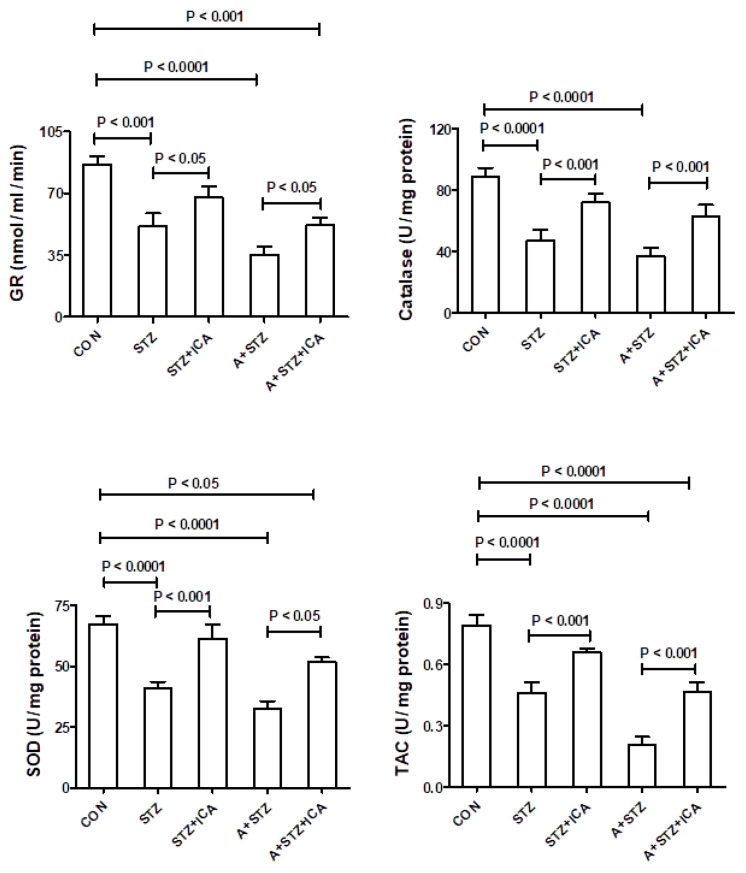
Effects of icariin (ICA) on the plasma concentration of superoxide dismutase (SOD) (U/mg protein), catalase (CAT) (U/mg protein), total antioxidant capacity (TAC) (U/mg protein), and glutathione reductase (GR) (nmol/mL/min) in control (CON) rats and rats treated with streptozotocin (STZ), with or without adenine (A).Values are presented as mean ± SEM for six animals per group (*n* = 6). The measurement units for each biomarker are indicated on the corresponding *y*-axes. Biomarker concentrations are expressed as units/mg protein, whereas enzyme activities are expressed as nmol/mL/min. Differences between groups were assessed using one-way analysis of variance (ANOVA), followed by Bonferroni’s multiple comparison test. Statistical significance was set at *p* < 0.05.

Both the diabetic group and diabetic CKD group showed significant changes in the inflammatory (Figure 2) and oxidative stress and defense markers (Figure 3 and Figure 4) compared to the control group. The diabetic group and diabetic CKD group both showed significant elevations in IL-6, IL-1β and TNF-α levels, and significantly lower levels of IL-10. Administration of ICA significantly improved these changes in both groups. The two experimental groups also had significantly higher 8-isoprostane, 8-OHDG and AGE levels, along with significantly lower GR activity and catalase, SOD and TAC levels. Treatment with ICA significantly ameliorated all of these perturbations in both experimental groups.

### 2.4. Histopathology

**Figure 5:**
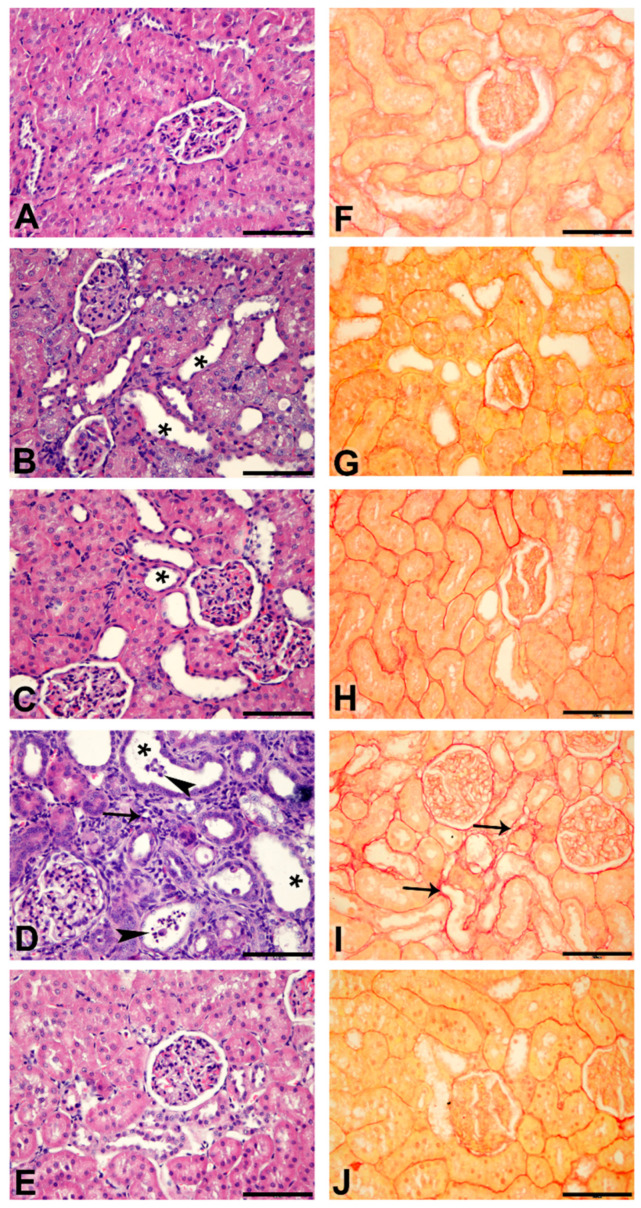
Photomicrographs of the renal cortex (Bar = 100 µm, (**A**–**E**): H&E; (**F**–**J**): Picrosirius Red). The control group exhibited normal histological renal structures with intact glomeruli and tubules (lesion score 0) (**A**,**F**). The streptozotocin (STZ)-treated group exhibited marked tubular dilatation with mild basophilia and intact glomeruli (lesion score 2) (**B**,**G**). The STZ + Icariin (ICA)-treated groups showed mild tubular dilatation with intact glomeruli (lesion score 2) (**C**,**H**). The adenine + STZ-treated group exhibited severe cystic tubular dilatation, basophilia of the renal tubules, numerous cellular casts, and interstitial nephritis (lesion score of 4) (**D**,**I**). The adenine + STZ + ICA group presented an intact glomerular tuft; most renal tubules had normal structures and some regenerating tubules (lesion score 1) (**E**,**J**). Asterisks denote cystic dilation of renal tubules, arrowheads point to cellular casts, and arrows indicate fibrosis.

The histopathological findings are presented in Figure 5 and Table 4. Histopathological evaluation of renal tissues from the control group revealed normal histological architecture, with intact glomeruli and tubules (lesion score 0). The STZ-treated group demonstrated marked tubular dilatation with mild basophilia and intact glomeruli (lesion score 2). The STZ + ICA-treated group displayed mild tubular dilatation with intact glomeruli (lesion score 2). The adenine + STZ-treated group exhibited severe cystic tubular dilatation, basophilia of the renal tubules, numerous cellular casts, and interstitial nephritis (lesion score 4). The adenine + STZ + ICA-treated group presented with intact glomerular tufts, where most renal tubules exhibited a normal structure, along with some regenerating tubules (lesion score 1).

## 3. Discussion

ICA is a pharmacologically active flavonoid extracted from the Chinese herbal plant *Epimedium brevicornum Maxim*. This is the first study to investigate the effects of 200 mg/kg of ICA in STZ-induced diabetic rats, either with or without adenine-induced CKD. STZ is a diabetogenic chemical that causes β-cell depletion, resulting in hyperglycemia, polyuria, weight loss, and other pathological features of DM [19]. The adenine-induced CKD rat model is widely recognized to mimic the disease trajectory of human CKD and to replicate its structural and functional manifestations [18]. These features of diabetes and kidney disease were replicated in the present study, where the experimental groups displayed hyperglycemia, weight loss, kidney hypertrophy, increased water intake and urine flow, along with impaired renal function, renal structural damage and dysregulated inflammatory and oxidative stress markers. As expected, these changes were more pronounced in the diabetic rats with CKD compared to those without CKD. Most of the biochemical aberrations and renal morphological changes observed in the experimental groups were significantly improved in the animals that received ICA.

Our findings support the blood glucose-lowering effects of ICA as demonstrated in previous experimental studies in rodent models of types 1 and 2 DM, metabolic syndrome, and insulin resistance [20,21,22,23]. According to findings from previous studies, ICA appears to exert its hypoglycemic effect through multiple mechanisms. One study showed that ICA preserves pancreatic islets and increases the protein and transcriptional gene expression of phospho-AMP-mediated protein kinase and glucose transporter isoform 4 [22]. Experimental evidence from another study also suggests that ICA acts as a peroxisome proliferator activated receptor-α activator that induces the transcription of various genes, such as CYP4A10 and CYP4A14, that are implicated in pathogenesis of insulin resistance [24,25]. Further evidence suggests that ICA promotes the release of insulin in response to glucose through actions mediated by the phosphatidylinositol 3-kinase/protein kinase B (PI3K/AKT) pathway [26]. Whether these pathways contribute to the metabolic effects observed in the present model remains to be determined and warrants further investigation. Despite mounting preclinical evidence to support its role in glycemic control and a favorable toxicity profile in humans [27], there are currently no published clinical trials investigating the safety and efficacy of ICA in patients with diabetes. Therefore, translational research is required to establish the clinical viability of ICA as a hypoglycemic agent in the management of diabetes.

The present study also provides evidence to support renoprotective properties of ICA. Our findings extend those of previous studies conducted in experimental rodents with unilateral obstruction, 5/6 nephrectomy-induced CKD, nephrotic syndrome, and STZ-induced diabetic kidney disease [12,15,28,29,30]. It is well established that the pathophysiological mechanisms of diabetes and CKD are closely interlinked, where progression of one leads to worsening of the other [31]. Hence, identifying pharmacological interventions that can mitigate renal disease progression while simultaneously maintaining glycemic control may help optimize clinical outcomes among patients with diabetes complicated by CKD. Here, we demonstrate the efficacy of ICA in ameliorating renal dysfunction in the context of diabetes, as well as in diabetes with CKD characterized by more severe markers of renal damage. Notably, we found that ICA-treated animals showed a marked reduction in renal fibrosis, with levels approaching normal, presenting with significant improvements in tubular necrosis scores and displaying signs of tubular regeneration. These findings are of major consequence, given that fibrosis in CKD is progressive in nature and, once advanced stages are established, can result in irreversible kidney failure [32]. The effect of ICA on tubular regeneration may be explained in light of its ability to stimulate the proliferation and differentiation of renal stem and progenitor cells via upregulation of genes such as Osr1, NMP-7, Pax2, and Wilms’ tumor-1 (WT-1), as demonstrated by Huang et al. [29]. In support of this, WT-1 is a transcription factor that is expressed in podocytes and is regulated by IL-1β and TNF-α [33], both of which were found to be strongly modulated by ICA in the present study.

The favorable effects of ICA on inflammation and redox imbalance may have further contributed to its renoprotective actions in the experimental groups. Indeed, chronic inflammation and oxidative stress, both exacerbated by a hyperglycemic state, are well established as the most critical pathological processes driving renal injury progression in diabetes [31]. Enhanced oxidative stress in CKD is thought to occur as a result of mitochondrial dysfunction coupled with increased mitochondrial generation of ROS [34]. The accumulation of uremic toxins as a result of CKD further exacerbates oxidative stress and contributes to the development and progression of inflammation [35]. In this study, ICA ameliorated perturbations in the pro-inflammatory and anti-inflammatory cytokines of the diseased models, reduced the levels of oxidative stress markers, and improved the levels and activity of antioxidant enzymes. Our results are consistent with those of other studies demonstrating the anti-inflammatory and antioxidative effects of ICA in a diverse range of cells and disease models, including vascular endothelial cells, intestinal epithelial cells, keratinocytes, cervical cancer cells, acute kidney injury and CKD, epilepsy, acute lung injury, inflammation, and atherosclerosis [13,16,36,37,38,39,40,41]. While the molecular mechanisms underlying the effects observed in the current study were not directly investigated, previous experimental studies have suggested that ICA may modulate several signaling pathways involved in inflammation and oxidative stress. Multiple studies have shown that ICA modulates PI3K/AKT and its downstream effector proteins [42,43]. The PI3K/AKT signal transduction pathway regulates β cell function, insulin secretion, glucose transport and uptake, and cellular metabolism, while also influencing processes implicated in CKD progression, such as inflammation, oxidative stress, apoptosis, autophagy, and epithelial–mesenchymal transition [44,45].

Numerous studies have further indicated that ICA influences the Wnt/β-catenin signaling pathway [46,47]. The nature of the interaction appears to be context-specific, where it has a stimulatory effect in some conditions and an inhibitory effect in others [48,49]. Some studies have reported that TNF-α mediates renal injury via the Wnt/β-catenin pathway [50]. Given that TNF-α was reduced by ICA in the present study, elucidating the effect of ICA on the Wnt/β-catenin pathway in the specific context of diabetes and CKD warrants further investigation.

Several limitations should be acknowledged. First, the absence of adenine-only CKD and ICA-only treatment groups precludes independent evaluation of the effects of adenine-induced CKD and ICA treatment alone. Second, the inclusion of only male animals limits generalizability of the findings to females. Third, as molecular analyses were not performed, the signaling pathways and mechanistic processes underlying the observed effects of ICA could not be directly evaluated. Fourth, histopathological evaluation was carried out by a single blinded observer, so interobserver agreement could not be determined. Finally, this study lacked pharmacokinetic assessments, preventing characterization of the relationship between ICA exposure and its pharmacological effects. Addressing these limitations will help provide a more comprehensive understanding of the mechanisms underlying the effects of ICA in diabetic CKD.

## 4. Materials and Methods

### 4.1. Animals

Male Wistar rats (200–300 g) were procured from the Small Animal House at Sultan Qaboos University. Animals were maintained under standardized laboratory conditions, including a temperature of 22 ± 2 °C, relative humidity of approximately 60%, and a 12 h light–dark cycle. Standard laboratory chow and tap water were provided ad libitum throughout the study period. The study was approved by the University Animal Ethics Committee, Sultan Qaboos University (Approval no. SQU/EC-AUR/2023-2024/8). All procedures involving animals and their care were carried out in compliance with international laws and policies.

### 4.2. Experimental Design

A total of thirty rats were used in this study and divided into five groups (n = 6 per group). The sample size was based on the Resource Equation method as described by Charan and Kantharia [51]. Animals were assigned to experimental groups based on their baseline body weight to achieve comparable weight distribution across groups. After one week of acclimatization, rats were treated for 35 consecutive days as follows:

Group 1 (Non-diabetic, non-CKD group, control): received normal feed and saline by gastric gavage.

Group 2 (Diabetic rats without CKD group, DM): received a single intraperitoneal injection of STZ (55 mg/kg) and saline by gastric gavage.

Group 3 (Diabetic rats without CKD treated with ICA group, DM + ICA): received a single intraperitoneal injection of STZ (55 mg/kg), and of ICA (200 mg/kg) by gastric gavage. ICA dose was selected based on a previous study demonstrating the efficacy of this dose in a CKD model [15].

Group 4 (Diabetic rats with experimentally induced CKD group, DM + CKD): received a single intraperitoneal injection of STZ (55 mg/kg), adenine (0.25% *w*/*w*) in the feed, and saline by gastric gavage.

Group 5 (Diabetic rats with experimentally induced CKD treated with ICA group, DM + CKD + ICA): received a single intraperitoneal injection of STZ (55 mg/kg), adenine (0.25% *w*/*w*) in the feed, and ICA (200 mg/kg) by gastric gavage.

One day before study termination, the animals were housed in individual metabolic cages to collect urine over a 24 h period. Upon completion of the treatment protocol, the animals were anesthetized as previously described [52]. Blood samples were obtained from the anterior vena cava, and plasma was separated using established protocols. The animals were sacrificed by an overdose of anesthesia. Plasma and urine samples were stored at −80 °C pending biochemical analysis. The kidneys and livers were excised, blotted dry on filter paper, and weighed. The right kidney and most of the left kidney were rapidly immersed in liquid nitrogen and kept frozen at −80 °C for further analysis. Representative portions of the left kidney and liver were fixed in 10% buffered formalin for histopathological examination.

### 4.3. Drugs and Chemicals

STZ and adenine were obtained from Sigma Aldrich (Saint Louis, MO, USA), and ICA was purchased from Zhishang Chemical Co., Ltd. (Jinan, China). Plasma concentrations of urea, creatinine, calcium, phosphorous, uric acid, and urine albumin were measured using a BS-120 automated chemistry analyzer (MINDRAY, Shenzhen, China). Urine N-acetyl-β-D-glucosaminidase (NAG), plasma neutrophil gelatinase-associated lipocalin (NGAL), cystatin C, interleukin-6 (IL-6), interleukin-1β (IL-1β), interleukin-10 (IL-10), 8-hydroxy-2′-deoxyguanosine (8-OHdG), and tumor necrosis factor alpha (TNF-α) were quantified using ELISA kits from Elabscience Bionovation Inc. (Houston, TX, USA). Plasma 8-isoprostane, advanced glycation end-products (AGEs), indoxyl sulphate (IS), and renalase were measured using ELISA kits from Assay Genie Ltd. (Windsor Place, Dublin, Ireland). Glutathione reductase (GR) was measured using colorimetric assay kits from Biovision (Milpitas, CA, USA). Super oxide dismutase (SOD), total antioxidant capacity (TAC), and catalase were measured using colorimetric assay kits purchased from Elabscience Bionovation Inc. (Houston, TX, USA). Urine osmolality was measured by freezing point osmometer (Genotec, GmbH, Berlin, Germany). These biomarkers were selected to capture complementary domains of renal injury. For example, cystatin C was measured as a marker of glomerular filtration that is independent of muscle mass, NGAL as an early indicator of tubular injury, IS as a representative protein-bound uremic toxin reflecting impaired renal clearance, and renalase as a kidney-derived enzyme involved in catecholamine metabolism and cardiovascular–renal regulation.

### 4.4. Histopathological Analysis

Kidneys were harvested at the end of the experiment and immersed in 10% neutral-buffered formalin for fixation. Paraffin-embedded renal tissue sections were routinely stained with Hematoxylin and Eosin (H & E) and Picrosirius Red (ab150681, Abcam, Cambridge, UK). Renal tubular necrosis was assessed blindly by a pathologist using a semi-quantitative scoring system ranging from 0 to 4, where 0 indicated the absence of necrosis, 1 represented involvement of less than 10% of the examined area, 2 corresponded to 10–25%, 3 to 26–75%, and 4 to more than 75% tubular necrosis [53]. Three 40X microscopic fields were analyzed from each kidney section of each animal of the five groups, and the score was calculated according to the mean percentage.

Fibrosis was assessed using the Picrosirius Red stain. Sirius Red-stained slides were evaluated as previously described by Manni et al., 2011 [54]. All slides, for both renal tubular necrosis scoring and fibrosis quantification, were evaluated by a single blinded observer; consequently, interobserver agreement was not assessed. The slides were microscopically examined using an Olympus BX51 microscope attached to an Olympus DP70 camera, and three random images of the renal cortex were acquired using the 40X objective lens. Images were acquired from each kidney of each animal in the five groups and stored as 24-bit RGB TIFF images. The same camera and microscope settings were used to capture all images. Image analysis was conducted using ImageJ^®^ image analysis software (http://rsbweb.nih.gov/ij/ (accessed on 1 December 2024)). Digital images were converted to grayscale, after which collagen-positive regions were isolated using hue histogram thresholding. Fibrotic deposition was quantified by calculating the proportion of Sirius Red-positive collagen staining relative to the total tissue area. The fibrosis index for each animal was expressed as the percentage of collagen-stained area averaged across the analyzed fields.

### 4.5. Statistical Analysis

Data were analyzed by one-way analysis of variance (ANOVA), followed by Bonferroni’s multiple comparison tests, and are presented as mean ± SEM. Owing to the small sample size per group (*n* = 6), formal tests for normality and homogeneity of variance were not performed. As all groups were compared with one another, Bonferroni correction was applied to control for multiple comparisons. A two-tailed *p* value < 0.05 was considered statistically significant. Statistical analyses were performed using GraphPad Prism version 5.03 (GraphPad Prism Software, San Diego, CA, USA).

## 5. Conclusions

ICA treatment in STZ-induced diabetic rodents—either with or without adenine-induced CKD—resulted in significant blood glucose control, improved kidney function and histological structure, reduced inflammation and oxidative stress, and enhanced antioxidant defenses. Despite strong evidence to support its safety and efficacy as a renoprotective agent across various disease models, there are currently no clinical trials investigating its clinical utility in diabetes and CKD. The results presented in this study further underscore the ameliorative effects of ICA in experimental CKD and diabetes and may provide a basis for future translational research in human subjects. Further mechanistic investigations and clinical studies are warranted to determine whether these findings translate to human disease.

## Figures and Tables

**Table 1:** Effect of Icariin (ICA) treatment on some physiological parameters in rats with both streptozotocin (STZ)-induced diabetes and adenine (A)-induced chronic kidney disease.

Parameters/ Treatments	Group 1 Control	Group 2 STZ	Group 3 STZ + ICA	Group 4 A + STZ	Group 5 A + STZ + ICA
Initial body weight (g)	273.2 ± 14.89	269.0 ± 4.80	271.5 ± 2.28	274.3 ± 15.5	269.3 ± 4.92
Final body weight (g)	334.17 ± 11.68	214 ± 8.69 ^a^	239.5 ± 11.32 ^a^	198.3 ± 15.29 ^a^	214.67 ± 9.21 ^a^
Body weight change (%)	23.75 ± 6.76	−20.22 ± 3.88 ^a^	−11.81 ± 3.95 ^a^	−27.74 ± 3.92 ^a^	−20.37 ± 2.54 ^a^
Relative kidney weight (%)	0.54 ± 0.01	0.84 ± 0.03 ^a^	0.90 ± 0.06 ^a^	1.01 ± 0.03 ^a^	0.91 ± 0.04 ^a^
Relative Liver weight (%)	2.79 ± 0.05	3.94 ± 0.21	3.42 ± 0.16	4.2 ± 0.11	4.09 ± 0.22
Water intake (mL)	17.17 ± 1.49	96.17 ± 2.85 ^a^	68.50 ± 9.74 ^a,b^	143.33 ± 12.69 ^a^	80.83 ± 6.86 ^a,c^
Urine flow (μL/min)	6.94 ± 0.95	62.27 ± 2.03 ^a^	36.69 ± 5.18 ^a,b^	74.42 ± 7.84 ^a^	43.75 ± 3.89 ^a,c^
Food intake (g)	19.17 ± 1.31	23.90 ± 1.37	24.22 ± 1.79	27.73 ± 2.61 ^a^	25.47 ± 2.08
Fecal output (g)	4.98 ± 0.81	10.82 ± 0.99	12.97 ± 2.08 ^a^	15.40 ± 1.33 ^a^	15.42 ± 1.44 ^a^

Values in the table are means ± SEM (*n* = 6). Statistical comparisons were performed using ANOVA, followed by Bonferroni’s multiple comparison test, where *p* < 0.05. ^a^ denotes significance of control group vs. all other groups. ^b^ denotes significance of STZ alone group vs. STZ + ICA-treated group. ^c^ denotes significance of A + STZ alone group vs. A + STZ + ICA-treated group.

**Table 2:** Effects of Icariin (ICA) treatment on plasma parameters in rats with both streptozotocin (STZ)-induced diabetes and adenine (A)-induced chronic kidney disease.

Parameters/ Treatments	Group 1 Control	Group 2 STZ	Group 3 STZ + ICA	Group 4 A + STZ	Group 5 A + STZ + ICA
Fasting blood sugar (mmol/L)	4.2 ± 0.11	29.68 ± 0.82 **^a^**	11.83 ± 2.43 **^a,b^**	32.7 ± 0.30 **^a^**	18.13 ± 2.42 **^a,c^**
Urea (mmol/L)	3.40 ± 0.43	8.38 ± 0.53 **^a^**	4.77 ± 0.48 **^b^**	11.84 ± 1.96 **^a^**	5.72 ± 0.28 **^c^**
Creatinine (µmol/L)	15.6 ± 1.49	30.70 ± 2.01 **^a^**	18.23 ± 1.43 **^b^**	52.1 ± 3.59 **^a^**	28.7 ± 2.36 **^a,c^**
Uric acid (µmol/L)	22.2 ± 0.41	30.25 ± 0.75	17.83 ± 1.48 **^b^**	58.4 ± 6.95 **^a^**	18.57 ± 2.34 **^c^**
Calcium (mmol/L)	0.76 ± 0.04	0.48 ± 0.02 **^a^**	0.62 ± 0.04 **^a,b^**	0.44 ± 0.03 **^a^**	0.57 ± 0.02 **^a,c^**
Phosphorous (mmol/L)	0.69 ± 0.06	0.97 ± 0.02 **^a^**	0.66 ± 0.04 **^b^**	1.44 ± 0.18 **^a^**	0.76 ± 0.08 **^c^**

Values in the table are means ± SEM (*n* = 6). Statistical comparisons were performed using ANOVA, followed by Bonferroni’s multiple comparison test, where *p* < 0.05. ^a^ denotes significance of control group vs. all other groups. ^b^ denotes significance of STZ alone group vs. STZ + ICA-treated group. ^c^ denotes significance of A + STZ alone group vs. A + STZ + ICA-treated group.

**Table 3:** Effects of Icariin (ICA) treatment on urine parameters in rats with both streptozotocin (STZ)-induced diabetes and adenine (A)-induced chronic kidney disease.

Parameters/ Treatments	Group 1 Control	Group 2 STZ	Group 3 STZ + ICA	Group 4 A + STZ	Group 5 A + STZ + ICA
Urine creatinine (µmol/L)	8514.0 ± 775.8	544.6 ± 31.5 ^a^	1783.7 ± 315.7 ^a,b^	364.7 ± 99.1 ^a^	960.1 ± 81.4 ^a,c^
Urine NAG (ng/mL)	3.71 ± 0.40	8.49 ± 0.81 ^a^	4.48 ± 0.57 ^b^	14.47 ± 1.30 ^a^	6.66 ± 0.87 ^c^
NAG creatinine ratio (ng/µmol)	0.47 ± 0.09	15.89 ± 1.75	2.90 ± 0.60	57.37 ± 15.75 ^a^	6.96 ± 0.94 ^c^
Albumin creatinine ratio (mg/μmol)	0.54 ± 0.08	2.36 ± 0.43 ^a^	1.23 ± 0.27 ^b^	2.43 ± 0.53 ^a^	1.88 ± 0.37
Creatinine clearance (mL/minute)	3.74 ± 0.41	1.13 ± 0.11 ^a^	3.25 ± 0.15 ^b^	0.49 ± 0.13 ^a^	1.54 ± 0.25 ^a,c^
Osmolality (mOsmol/kg)	1710 ± 142.7	882 ± 33.04 ^a^	1355 ± 54.13 ^a,b^	656 ± 37.7 ^a^	1145 ± 36.19 ^a,c^

Values in the table are means ± SEM (*n* = 6). Statistical comparisons were performed using ANOVA, followed by Bonferroni’s multiple comparison test, where *p* < 0.05. ^a^ denotes significance of control group vs. all other groups. ^b^ denotes significance of STZ alone group vs. STZ + ICA-treated group. ^c^ denotes significance of A + STZ alone group vs. A + STZ + ICA-treated group.

**Table 4:** Lesion scores and fibrosis indices of renal tissues from the different treatment groups.

Group	Lesion Score	Fibrosis Index %
Control	0	5.6 ± 0.10
STZ	2	7.0 ± 0.95
STZ + ICA	2	6.0 ± 0.32
Adenine + STZ	4	20.2 ± 1.38 **^a,b,c^**
Adenine + STZ + ICA	1	5.9 ± 0.16 ^d^

Fibrosis index values are expressed as mean ± SEM (*n* = 6). Statistical comparisons were performed using ANOVA, followed by Bonferroni’s multiple comparison test. The fibrosis index was significantly higher in the adenine + streptozotocin (STZ) group compared with the control group (*p* < 0.05), and it was significantly reduced in the adenine + STZ + Icariin (ICA) group compared with the adenine + STZ group (*p* < 0.05). ^a^ denotes significance of control group vs. all other groups. ^b^ denotes significance of STZ alone group vs. adenine + STZ-treated group. ^c^ denotes significance of STZ + ICA alone group vs. adenine + STZ-treated group. ^d^ denotes significance of adenine + STZ alone group vs. adenine + STZ + ICA-treated group.

## Data Availability

The original contributions presented in this study are included in the article. Further inquiries can be directed to the corresponding author.
